# Supplementary material for: MetaRibo-Seq measures translation in microbiomes
Source: Nat Commun. 2020 Jun 29;11:3268. doi: 10.1038/s41467-020-17081-z (PMC7324362; doi:10.1038/s41467-020-17081-z)
Supplement: Supplementary file 10 — Supplementary Data 7 [file 41467_2020_17081_MOESM10_ESM.zip › File2/Confidence_High_Taxonomy/337840_out.krona.html]

Javascript must be enabled to view this page.

members
magnitude
magnitudeUnassigned
count
unassigned
taxon
rank

337840\_out

6

superkingdom
6
2

1239
6
phylum

class
186801
6

order
186802
6

family
6
424536

genus
6
270497


SRS020233\_contig\_number\_contig-100\_27272.153368SRS024075\_contig\_number\_9275SRS054352\_contig\_number\_contig-100\_576.125993SRS056273\_contig\_number\_contig-100\_16241.52942SRS077392\_contig\_number\_contig-100\_6088.163063SRS147271\_contig\_number\_31780
1946296
6
species
